# A *Picea crassifolia* Tree-Ring Width-Based Temperature Reconstruction for the Mt. Dongda Region, Northwest China, and Its Relationship to Large-Scale Climate Forcing

**DOI:** 10.1371/journal.pone.0160963

**Published:** 2016-08-10

**Authors:** Yu Liu, Changfeng Sun, Qiang Li, Qiufang Cai

**Affiliations:** 1 Department of Earth and Environmental Science, School of Human Settlements and Civil Engineering, Xi'an Jiaotong University, Xi'an, 710049, China; 2 The State Key Laboratory of Loess and Quaternary Geology, Institute of Earth Environment, Chinese Academy of Sciences, Xi'an, 710061, China; 3 Joint Center for Global Change Studies (JCGCS), Beijing Normal University, Beijing, 100875, China; Universidade de Vigo, SPAIN

## Abstract

The historical May–October mean temperature since 1831 was reconstructed based on tree-ring width of Qinghai spruce (*Picea crassifolia* Kom.) collected on Mt. Dongda, North of the Hexi Corridor in Northwest China. The regression model explained 46.6% of the variance of the instrumentally observed temperature. The cold periods in the reconstruction were 1831–1889, 1894–1901, 1908–1934 and 1950–1952, and the warm periods were 1890–1893, 1902–1907, 1935–1949 and 1953–2011. During the instrumental period (1951–2011), an obvious warming trend appeared in the last twenty years. The reconstruction displayed similar patterns to a temperature reconstruction from the east-central Tibetan Plateau at the inter-decadal timescale, indicating that the temperature reconstruction in this study was a reliable proxy for Northwest China. It was also found that the reconstruction series had good consistency with the Northern Hemisphere temperature at a decadal timescale. Multi-taper method spectral analysis detected some low- and high-frequency cycles (2.3–2.4-year, 2.8-year, 3.4–3.6-year, 5.0-year, 9.9-year and 27.0-year). Combining these cycles, the relationship of the low-frequency change with the Pacific Decadal Oscillation (PDO), North Atlantic Oscillation (NAO) and Southern Oscillation (SO) suggested that the reconstructed temperature variations may be related to large-scale atmospheric-oceanic variations. Major volcanic eruptions were partly reflected in the reconstructed temperatures after high-pass filtering; these events promoted anomalous cooling in this region. The results of this study not only provide new information for assessing the long-term temperature changes in the Hexi Corridor of Northwest China, but also further demonstrate the effects of large-scale atmospheric-oceanic circulation on climate change in Northwest China.

## Introduction

The Hexi Corridor is the most important grain production base and cash crop area in Northwest China [1]. Under the background of global warming, the temperature of the Hexi Corridor is unusually warm, resulting in deterioration of the ecological environment and transformation of the layout and structure of agricultural production [2]. The northern and southern mountains of the Hexi Corridor play an important role in the ecology and climate change of the corridor and its surrounding areas [3]. Therefore, it is obviously significant to understand the characteristics of climatic change with respect to these mountains. However, the instrumental data around the corridor feature only short time records that are insufficient for capturing the regularity and mechanisms of climate; therefore, paleoclimatic studies are critically important [4–6].

With its precise dating, high continuity, high resolution and easily obtained duplicates, the dendroclimatology approach is significant in studying paleoclimatic changes [7–11]. In recent decades, dendroclimatology has achieved important development in China [12–22]. There have been some climate reconstructions based on tree-ring indices in the Hexi Corridor and its vicinity, but these studies were mainly confined to the southern mountains, such as the Qilian Mountains, and most studies mainly focused on precipitation reconstruction [23–27]. Only one precipitation series was reconstructed using tree-ring indices in the northern mountainous region [28]. It remains unclear whether the trees in the northern mountains only reflect the precipitation signal and whether the climate change is similar to that in the southern mountains. Additionally, the question of whether the regional climate change regime around the Hexi Corridor is consistent on a long-term scale also remains unresolved. Therefore, it is essential to perform more dendroclimatological studies in the northern mountains of the Hexi Corridor. In this research, we selected Qinghai spruce (*Picea crassifolia* Kom.) from Mt. Dongda, which is located to the north of the Hexi Corridor, to build a tree-ring chronology for Mt. Dongda, to investigate the climatic response of the chronology and reconstruct the temperature since 1831, and to reveal the connections of the reconstructed temperature to large-scale climate forcing, including the Southern Oscillation (SO), North Atlantic Oscillation (NAO), Pacific Decadal Oscillation (PDO) and major volcanic eruptions.

## Materials and Methods

### Site description and tree-ring data

**Fig 1:**
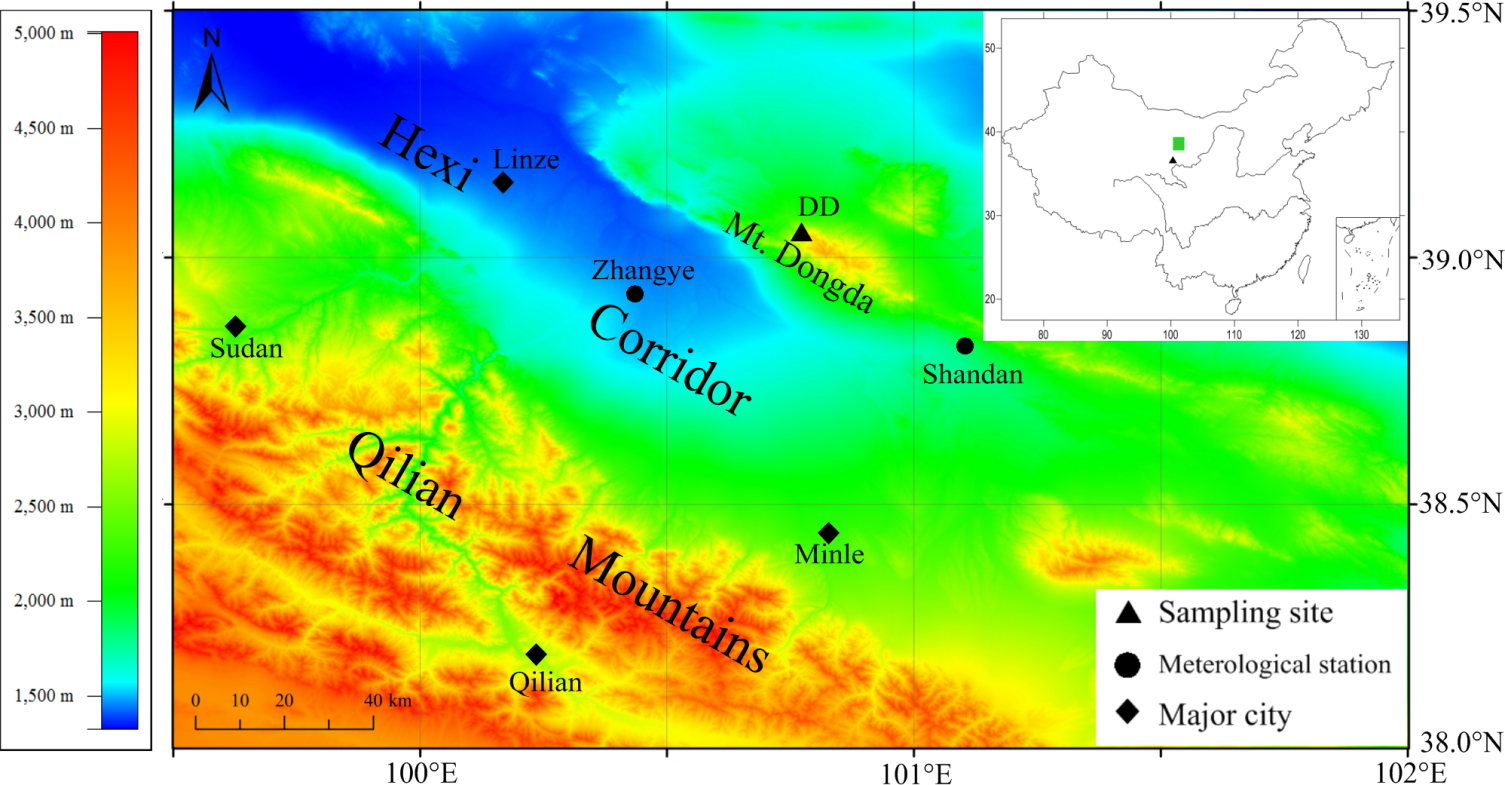
Location map of the tree-ring sampling sites and meteorological stations. The black triangle in the small picture in the upper right is the site used for comparison in this study.

With the permission of the Nature Reserve Station of the Dongda Mountains, samples were collected from Mt. Dongda (39°03′N, 100°46′E), located to the North of the Hexi Corridor (Fig 1). In this region, the mean annual temperature is 4.9°C, and the annual precipitation varies widely at different altitudes. The annual precipitation is approximately 150–190 mm at elevations of less than 2200 m and 300–400 mm at elevations of 2400–3600 m [29]. Because Qinghai spruce is an ombrophyte, we selected sampling sites on the northern slopes at elevations between 2900 and 3200 m. The soils of the sites are mainly mountain gray cinnamon soils that have a high water content. The vegetation coverage (shrubs and trees) is greater than 0.8, and the dominant tree species is Qinghai spruce. To minimize non-climatic effects on tree growth, only healthy trees with little evidence of fire or human disturbance were selected. A total of 66 cores from 36 living trees were ultimately sampled using 5-mm increment borers. The group of samples was named DD.

### Chronology development

In the laboratory, all the tree-ring cores were naturally dried, mounted, surfaced, and cross-dated following standard dendrochronological procedures [30]. The quality of cross-dating was controlled using the COFECHA program [31] (http://www.ldeo.columbia.edu/tree-ring-laboratory/resources/software). Cores with ambiguous results and those that were too short were excluded from further analysis, and 60 cores from 35 trees were used to establish the chronology. The results from the COFECHA program showed that the mean correlation coefficient of each series with a master series was 0.66 and the mean sensitivity was 0.21.These data indicated that the ring width variation pattern of each series was relatively similar.

**Fig 2:**
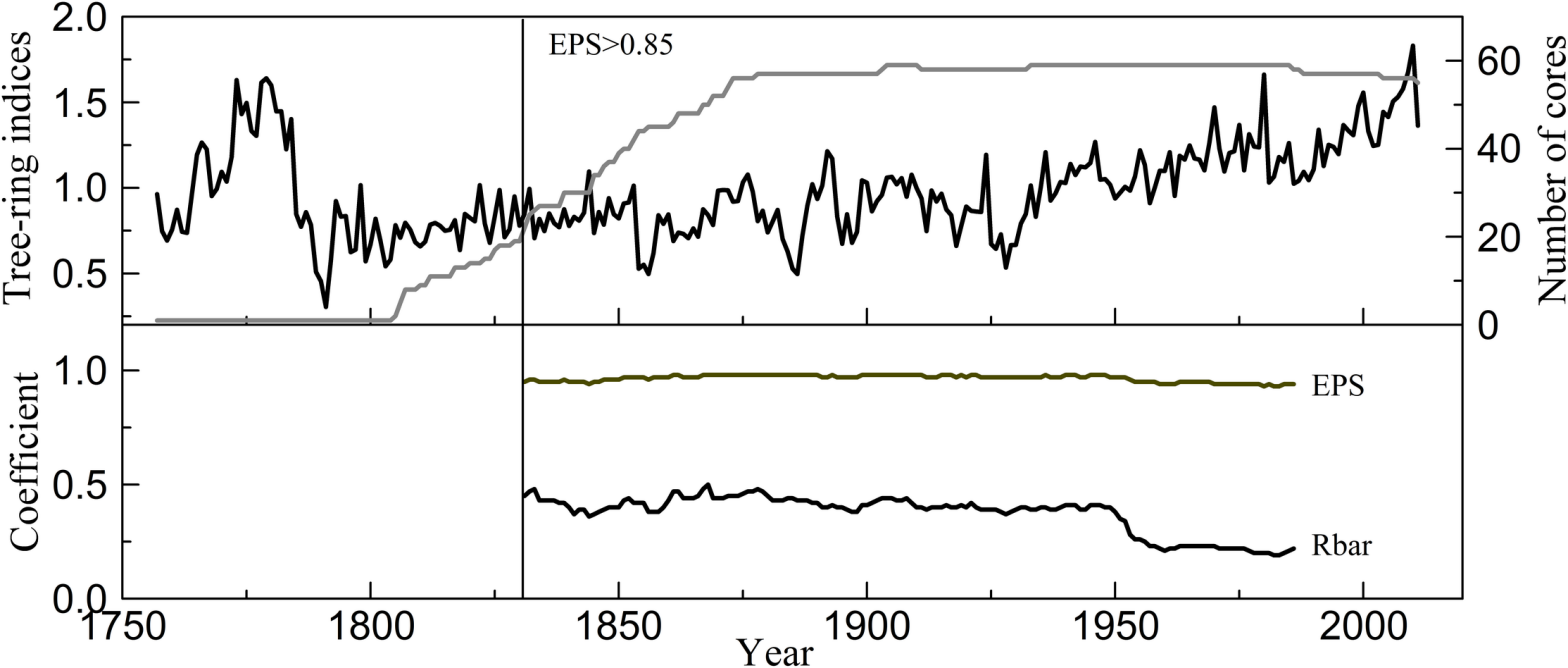
Tree-ring STD chronology and sample size and the running EPS and Rbar.

The CRUST program [32] (http://www.ldeo.columbia.edu/tree-ring-laboratory/resources/software) was used for detrending and developing of the tree-ring chronologies with the selection of “curve-fit detrend”. Three types of chronologies could be obtained: standardized (STD), residual (RES), and autoregressive standardized (ARS). In further analysis, the STD chronology was used because it preserved both low- and high-frequency information. The expressed population signal (EPS) can be used to evaluate the reliability of the tree-ring chronology [33]. In general, the greater the sample size, the higher the EPS values. An EPS value above 0.85 is generally regarded as satisfactory [34]. In this research, an EPS value exceeding 0.85 existed after AD 1831 and corresponded to 12 trees (Fig 2).

### Meteorological data

**Fig 3:**
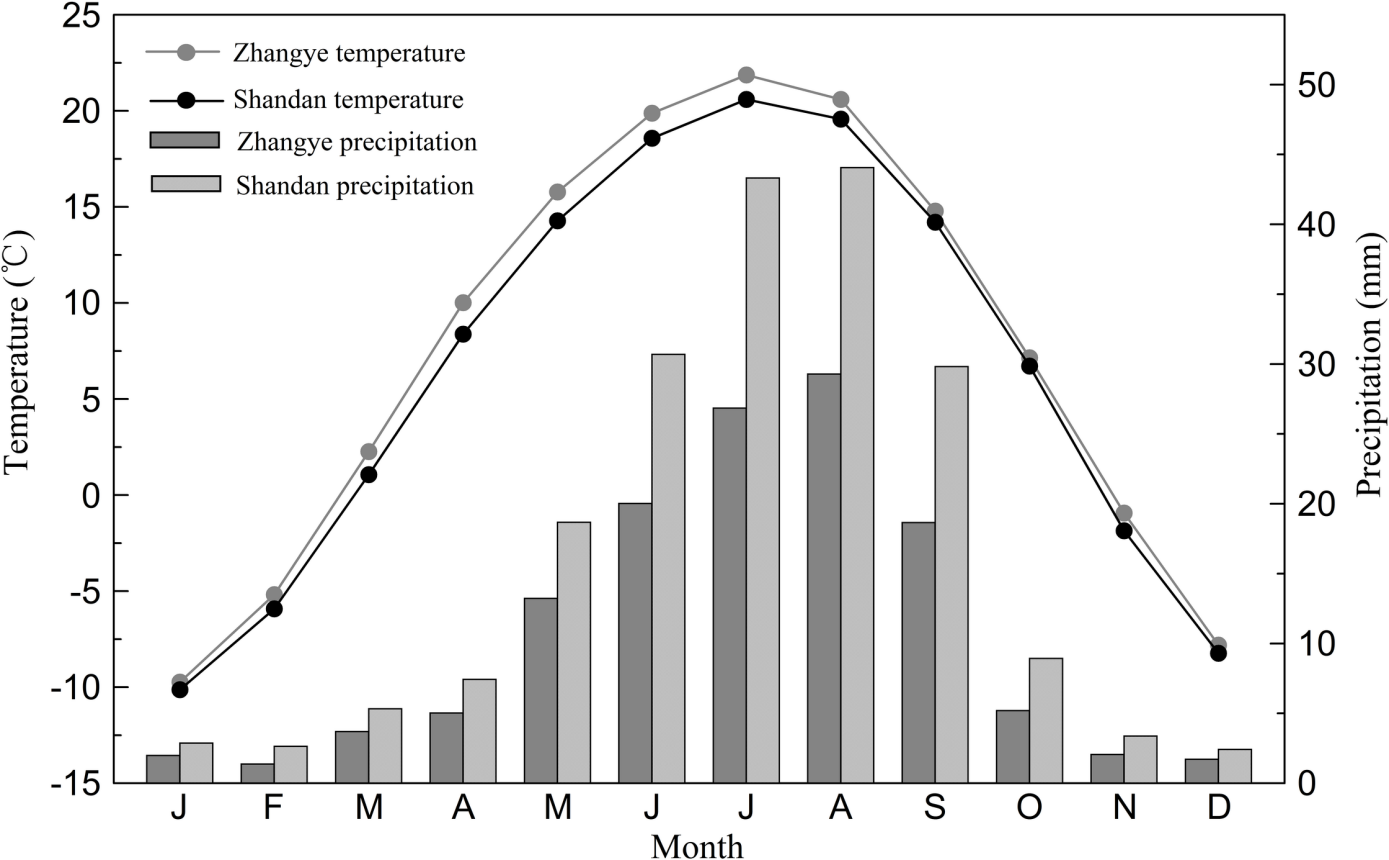
Monthly mean temperature and total precipitation at Zhangye (1951–2011) and Shandan (1953–2011) meteorological stations.

There are two meteorological stations near the sampling site: Zhangye (38°56′N, 100°26′E; elevation 1483 m; covering the period of 1951–2011) and Shandan (38°48′N, 101°05′E; elevation 1765 m; covering the period of 1953–2011). The monthly mean temperature and total precipitation of each station are shown in Fig 3. The monthly variation patterns for the two climate factors were almost synchronous between the two meteorological stations. Preliminary analyses showed that random errors and obvious uneven distributions for the climate data were not present in the meteorological records, thereby revealing that the records were reliable. Because the Zhangye meteorological station is closer to the sampling site and has a relatively long record period, the climatic data from this station were used for further analyses in this paper.

### Statistical methods

In this paper, Pearson's correlation analyses were used to identify climate-growth relationships between the tree-ring width indices and climate data from the observation period. Then, a simple linear regression model was used for temperature reconstruction. The bootstrap and jackknife [35] statistical methods, which have been employed in dendroclimatology [36–38], were used to check the stability of the regression model. The concept behind the bootstrap resampling technique is that the available observations of a variable contain the necessary information to construct an empirical probability distribution of any statistic of interest. The bootstrap method can provide standard errors of statistical estimators even when no theory exists. The jackknife method involves calculating the correlation of the time series after progressively removing the values for one year throughout the entire time period. Multi-taper method spectral analysis [39] was conducted to identify the periodicity in the reconstructed series. Superposed epoch analysis (SEA) [40] and the Monte Carlo test [41] were used to discuss the teleconnection between our reconstructed temperature and volcanic eruptions.

## Results

### Tree-ring climatic response

**Fig 4:**
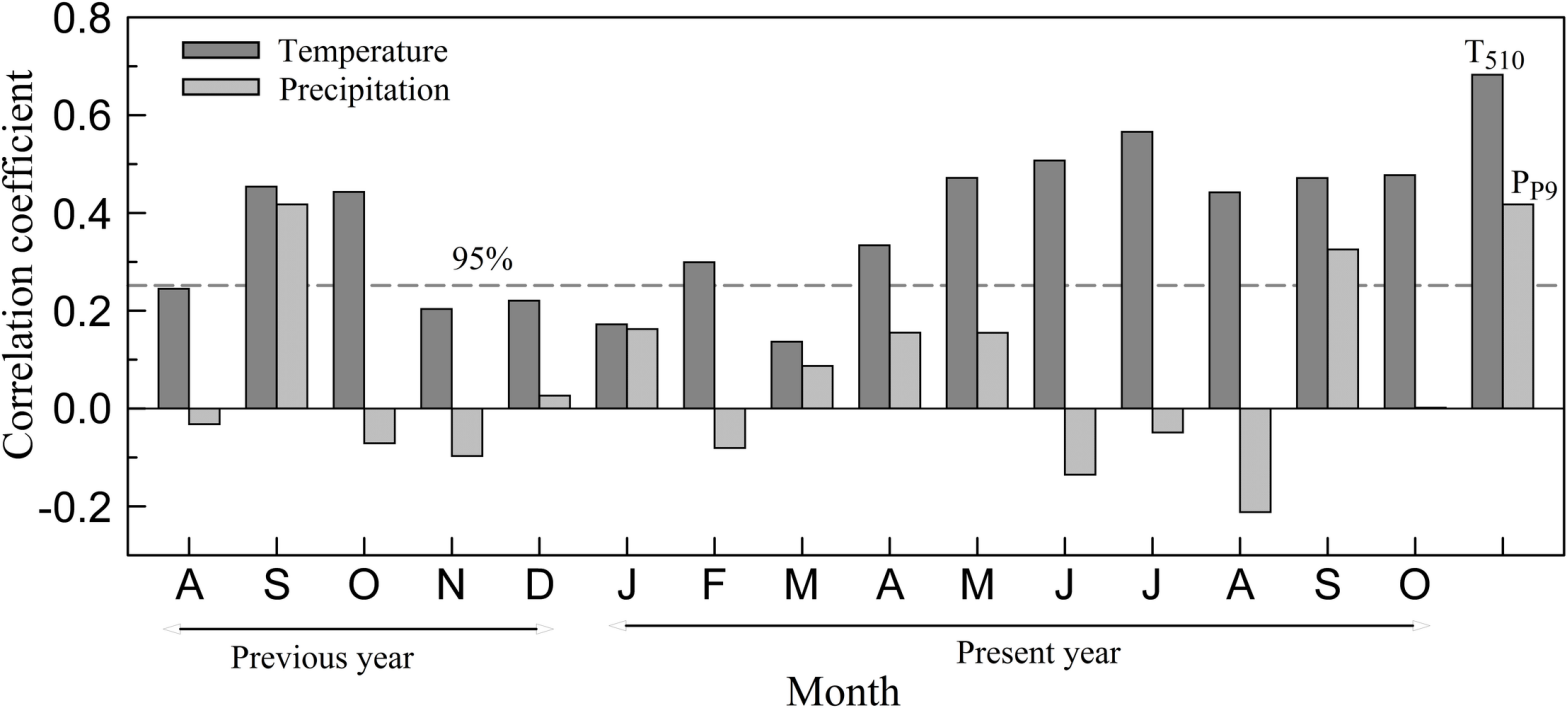
Correlation between tree-ring chronology and climatic records for 1951–2011. T_510_ is the mean temperature from May to October of the present year. P_P9_ is the monthly precipitation of the previous September.

Because the climate of the previous year may affect tree growth in the present year [42], the response analyses were assessed using the recorded climatic variables from August of the previous year to October of the present year (Fig 4). The correlation function results showed that the DD ring width had a significant correlation with precipitation in the previous and present September. In contrast, the correlations between temperature and tree growth were significantly positive and exceeded the 95% significance level for most months. Generally, seasonal climate is more meaningful and stable than that of a single month. After combining the monthly data on all aspects of the climatic conditions, the months with the highest correlations for the mean temperature were those from May to October (r = 0.683) (Fig 4). The months from May to October are close to the growing season (from June to early October) of Qinghai spruce in the Mt. Dongda area [43]. During these months, higher temperatures may promote photosynthesis and generate larger rings. Based on the analyses above, it made sense that the May–October mean temperature was the limiting factor for the radial width of Qinghai spruce growing on Mt. Dongda. This result suggested that the climate signal contained in the tree-ring width from the northern mountains was different from that of the southern mountains [23–27]. This was likely because of the different climates between these mountains. Based on the same tree species and a similar sampling site, it has been previously found that precipitation was the dominant factor controlling Qinghai spruce growth on Mt. Dongda [28], which was different from our result. This was likely because RES chronology was used in that study. It is known that RES chronology conserves much higher frequency signals [34] and is usually used to reconstruct past hydroclimate changes [23, 28, 44–46].

### Transfer function

Based on the above analyses, the temperature from May to October was reconstructed from the DD tree rings using the following linear regression model: T_510_ = 2.799*W_t_+13.163, (r = 0.683; R^2^ = 0.466; R^2^_adj_ = 0.457; F = 51.465; p<0.0001; D/W = 1.202), where T_510_ is the mean temperature from May to October (1951–2011), and W_t_ represents the index of the STD tree-ring width chronology.

**Fig 5:**
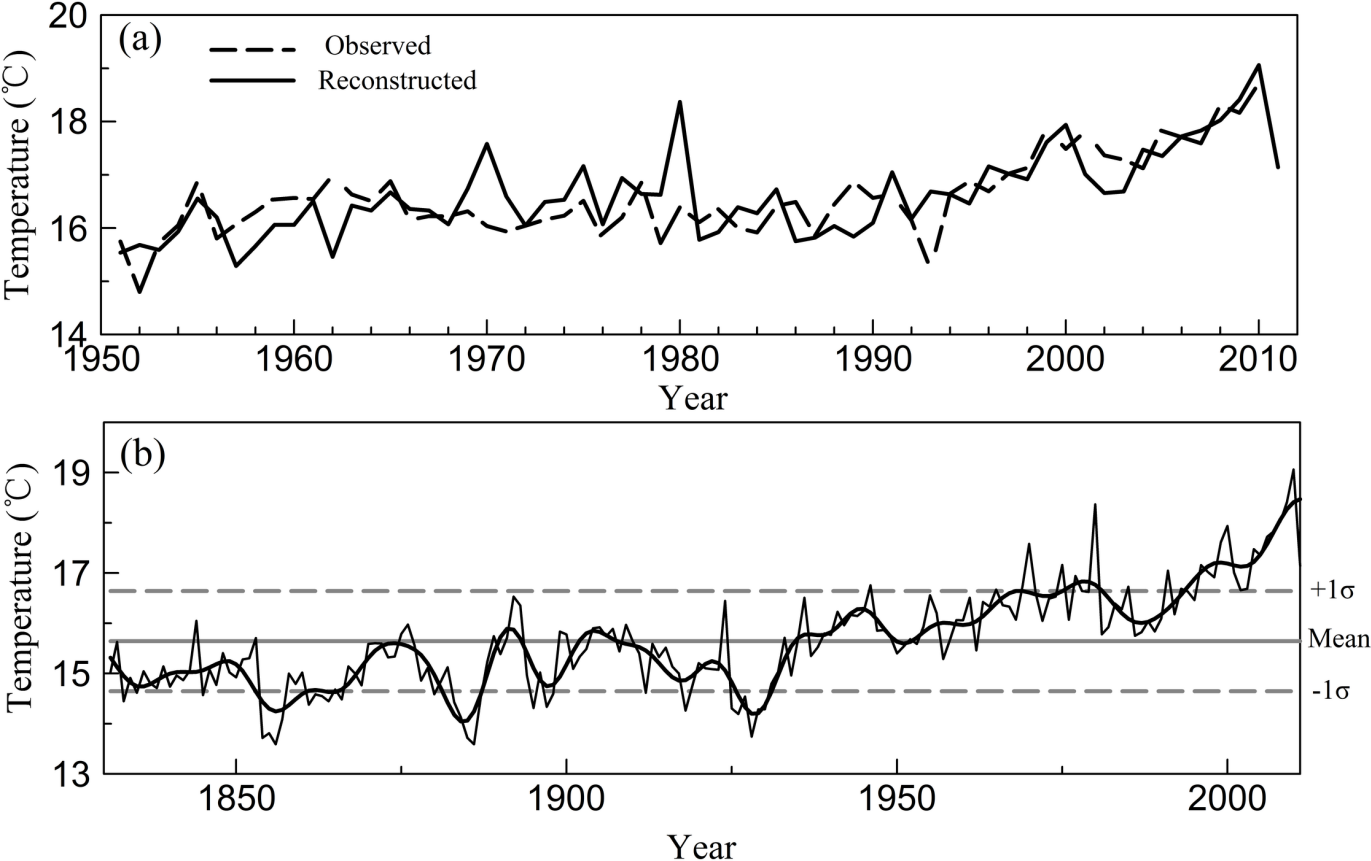
The observed and reconstructed May–October mean temperature (T_510_). (a) Comparison between observed and reconstructed T_510_ for their common period of 1951–2011 and (b) T_510_ reconstruction for Mt. Dongda since 1831. The thick line in (b) shows the smoothed data with a 10-year low-pass filter. The solid horizontal line represents the long-term mean for the period of 1831–2011; the dashed horizontal lines represent the mean value ± 1σ.

The test results of the robustness and reliability of the relationship between the chronology and the May–October mean temperature are shown in Table 1. Both the bootstrap and jackknife methods generated similar statistical results for the calibration period, suggesting that the temperature reconstruction was stable and reliable. In addition, Fig 5A showed that the reconstruction closely tracked the observed temperature.

**Table 1:** Verification results of the bootstrap and jackknife methods for May–October temperature reconstruction.

Statistical items	Calibration period (1951–2011)	Verification period (1951–2011)	
		Bootstrap (100 iterations) mean (range)	Jackknife mean (range)
r	0.683	0.67 (0.47–0.84)	0.68 (0.63–0.73)
R^2^	0.466	0.46 (0.22–0.71)	0.47 (0.40–0.53)
R^2^_adj_	0.457	0.45 (0.21–0.70)	0.46 (0.39–0.52)
SE	0.581	0.57 (0.40–0.72)	0.58 (0.55–0.58)
F	51.465	54.46 (17.04–142.38)	50.69 (38.66–64.23)
P	<0.0001	<0.0001	<0.0001
D/W	1.202	2.01 (1.18–2.60)	1.21 (1.11–1.38)

### May–October mean temperature reconstruction

Using the above transfer function, we reconstructed the May–October mean temperature history since 1831 (Fig 5B, see data in S1 Text). During the reconstructed interval (1831–2011), the mean T_510_ was 15.64°C, and the standard deviation (σ) was 1.00°C.

### Periodicity analysis for the reconstructed temperature

**Fig 6:**
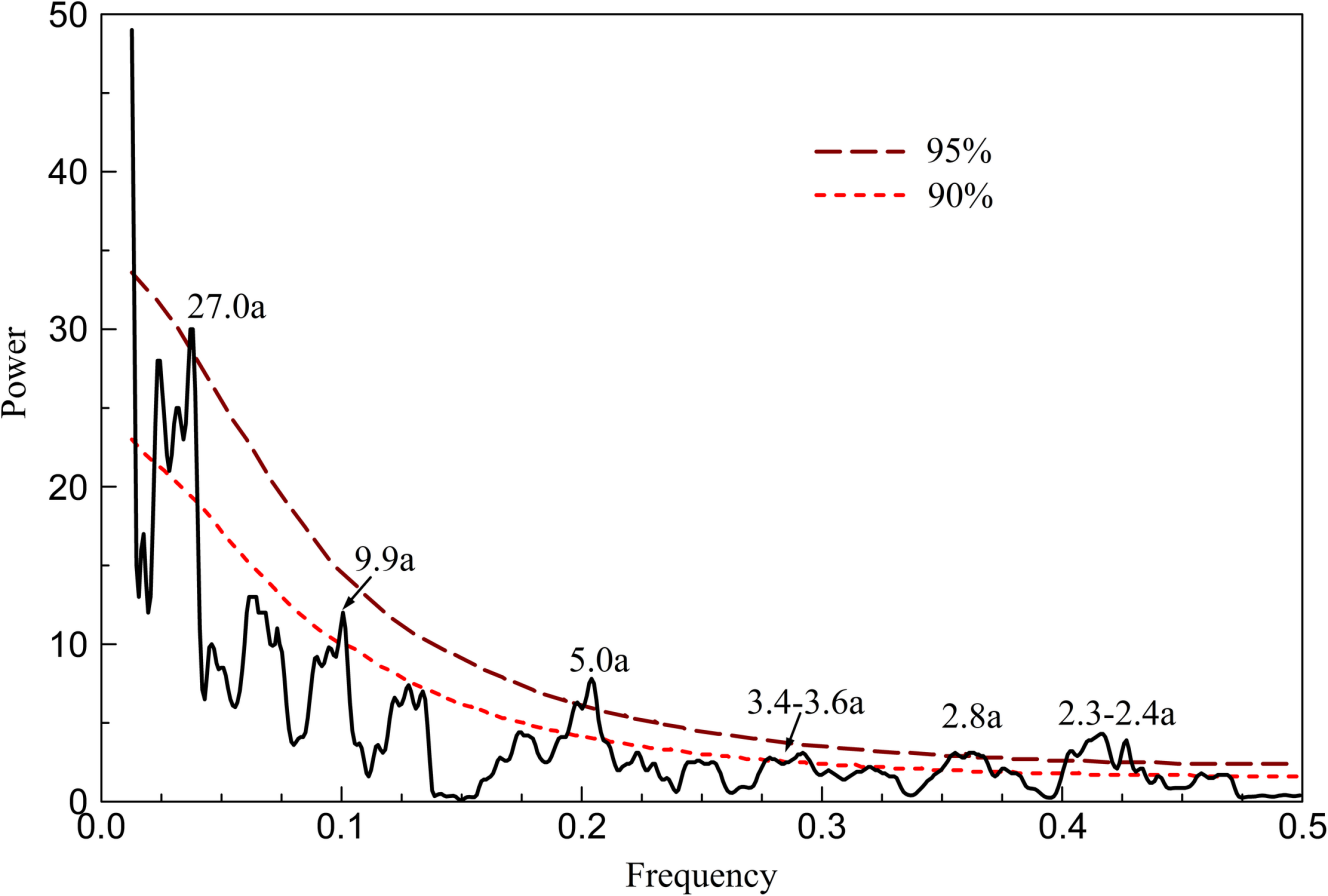
Spectrum analysis result of the reconstructed mean temperature. The dashed line indicates the 95% and 90% confidence levels.

Multi-taper method spectral analysis [39] was conducted to identify the periodicities in the reconstructed series. The results showed that the reconstructed temperature for Mt. Dongda contained significant periodicities at 2.3–2.4-year, 2.8-year, 3.4–3.6-year, 5.0-year, 9.9-year and 27.0-year (Fig 6).

## Discussion

### May–October mean temperature variations

To investigate historical temperature changes, we defined a warm year as >mean+1σ (16.64°C) and a cold year as <mean−1σ (14.64°C). The full reconstruction of the mean temperature displayed strong interannual variability from 1831 to 2011. During the last 181 years, 27 warm years and 29 cold years occurred in the reconstruction series. These warm and cold years accounted for 14.92% and 16.02% of the total, respectively. The twenty warmest/coldest years, with their T_510_ values, are shown in Table 2.

**Table 2:** The twenty warmest/coldest years in the reconstructed May–October mean temperature series (T_510_).

Rank	Warmer year	T_510_ (°C)	Colder year	T_510_ (°C)
1	2010	19.06	1856	13.59
2	2009	18.41	1886	13.59
3	1980	18.37	1854	13.72
4	2008	18.02	1885	13.72
5	2000	17.94	1928	13.74
6	2007	17.83	1855	13.81
7	2006	17.72	1857	14.09
8	1999	17.61	1884	14.13
9	1970	17.58	1926	14.19
10	2004	17.47	1918	14.26
11	2005	17.35	1929	14.28
12	1975	17.16	1930	14.29
13	1996	17.16	1925	14.30
14	2011	17.14	1895	14.31
15	1991	17.05	1897	14.34
16	1997	17.02	1861	14.38
17	2001	17.01	1883	14.43
18	1977	16.94	1833	14.45
19	1998	16.92	1864	14.45
20	1946	16.76	1866	14.48

To analyze the low-frequency variation of the reconstruction, a 10-year low-pass filter was applied (Fig 5B). After smoothing, the entire reconstructed temperature curve presented four warm periods (1890–1893, 1902–1907, 1935–1949 and 1953–2011) with temperatures higher than the mean, and four cold periods (1831–1889, 1894–1901, 1908–1934 and 1950–1952) with temperatures lower than the mean. Among them, the interval 1953–2011 was the longest warm period, and 1831–1889 was the longest cold period in the reconstructed period. There was an obvious warming trend in the last twenty years. In addition, this warming trend has also been observed widely in other dendroclimatological studies in China [47–50]. The 10-year low-pass filtering series also showed that there were three colder periods (1853–1860, 1882–1887, 1926–1931) with temperatures lower than the mean−1σ and two warmer periods (1975–1981, 1994–2011) with temperatures higher than the mean+1σ.

### Comparison with other tree-ring-based temperature reconstructions

**Fig 7:**
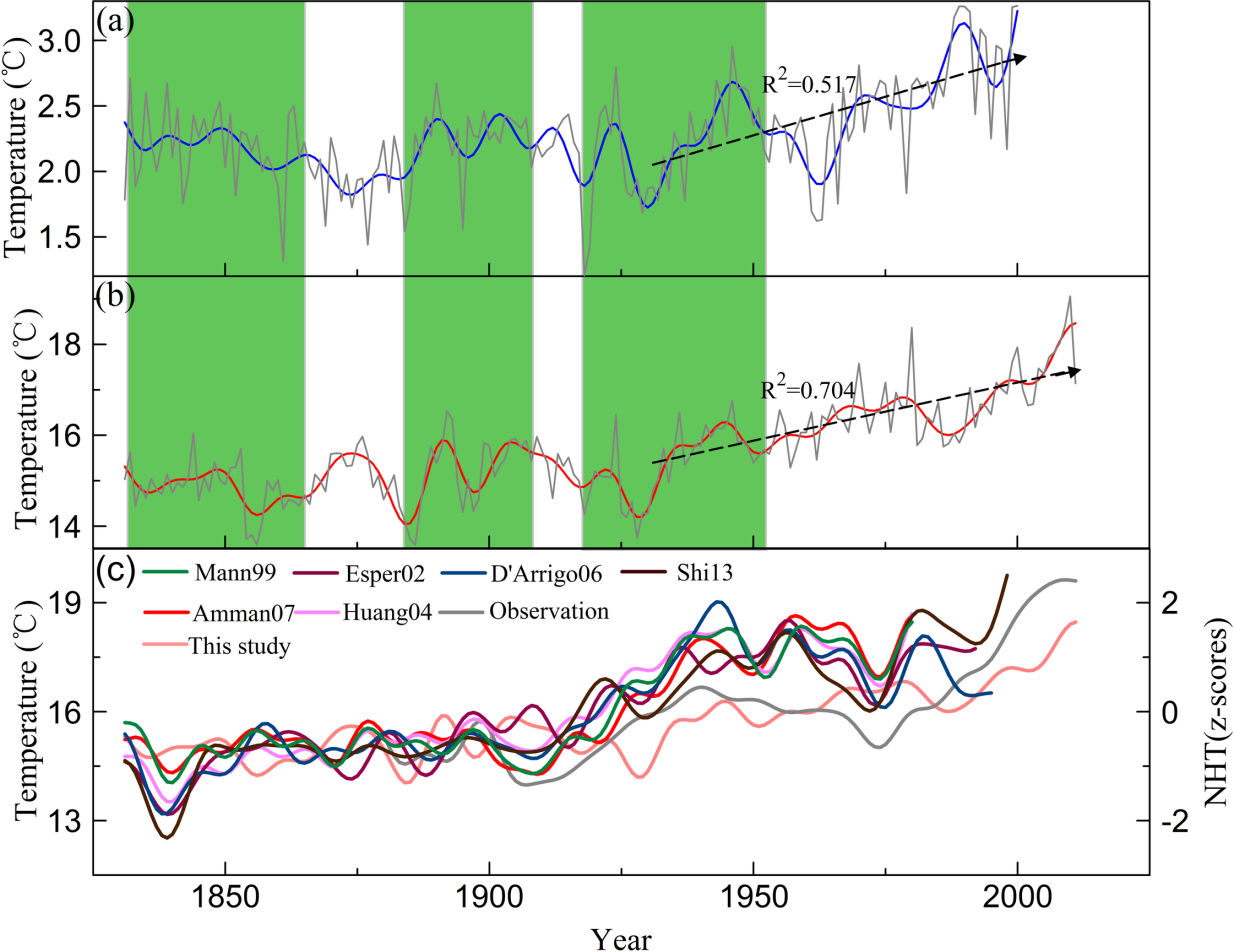
**Comparison between the mid-eastern Tibetan Plateau temperature (a), the reconstructed temperature (b), and the NH temperature (c).** All time series were smoothed using a 10-year low-pass filter. Vertical bars in (a) and (b) indicate when the two temperature series are in-phase, and the dashed line with an arrow indicates the linear fitting values during the period 1931–2011.

The reconstructed temperature series in this study provides useful information for analyzing climate change in the Hexi Corridor. Due to the lack of temperature reconstructions from areas around the research region, we compared our reconstruction with a tree-ring-based temperature reconstruction from Wulan-Dulan, in the east-central Tibetan Plateau [16, 51], to evaluate the reliability of the reconstruction. Both series were smoothed using a 10-year low-pass filter (Fig 7A and 7B). The reconstructed temperature series for Mt. Dongda correlates well with the temperature series of Wulan-Dulan with r = 0.55 (p<0.001, n = 170) on an annual scale and r = 0.67 (p<0.01, n = 17) on a decadal scale. Comparisons between these series revealed that the warm/cold variations were coherent with the low-frequency data at the large scale. These two series contained the same cooling/warming periods. Starting in the 1930s, both series exhibited a significant warming trend. Of course, there were nonconformities in these series during some periods, which may have been caused by possible differences in microhabitats at the sampling site and/or the reconstruction of different seasonal periods. On the whole, the results indicated that the Mt. Dongda tree-ring index reflected the temperature history not only of the study area, but also of the larger region of Northwest China.

Further analysis indicated that our reconstructed temperature exhibited changes on the long-term scale that are similar to those of the Northern Hemisphere (NH) temperature. The reconstructed temperature was significantly correlated with the NH temperature reconstruction [10, 52–56]. The correlation coefficients ranged from 0.44 to 0.59 (p<0.001, n>150). In addition, there was also a remarkable relationship between our temperature and the NH mean May–October temperature observations (r = 0.60, 1880–2011, p<0.0001). After application of a 10-year low-pass filter, the low-frequency changes appeared to be coherent among these temperature series (Fig 7C). These findings indicated that there was good consistency between the temperature of Mt. Dongda and the NH temperature at a low-frequency change and also suggested that our reconstructed series could be used as a proxy for other researchers to reconstruct large-scale regional temperature, such as the NH temperature.

### Teleconnections with SO, PDO and NAO

**Fig 8:**
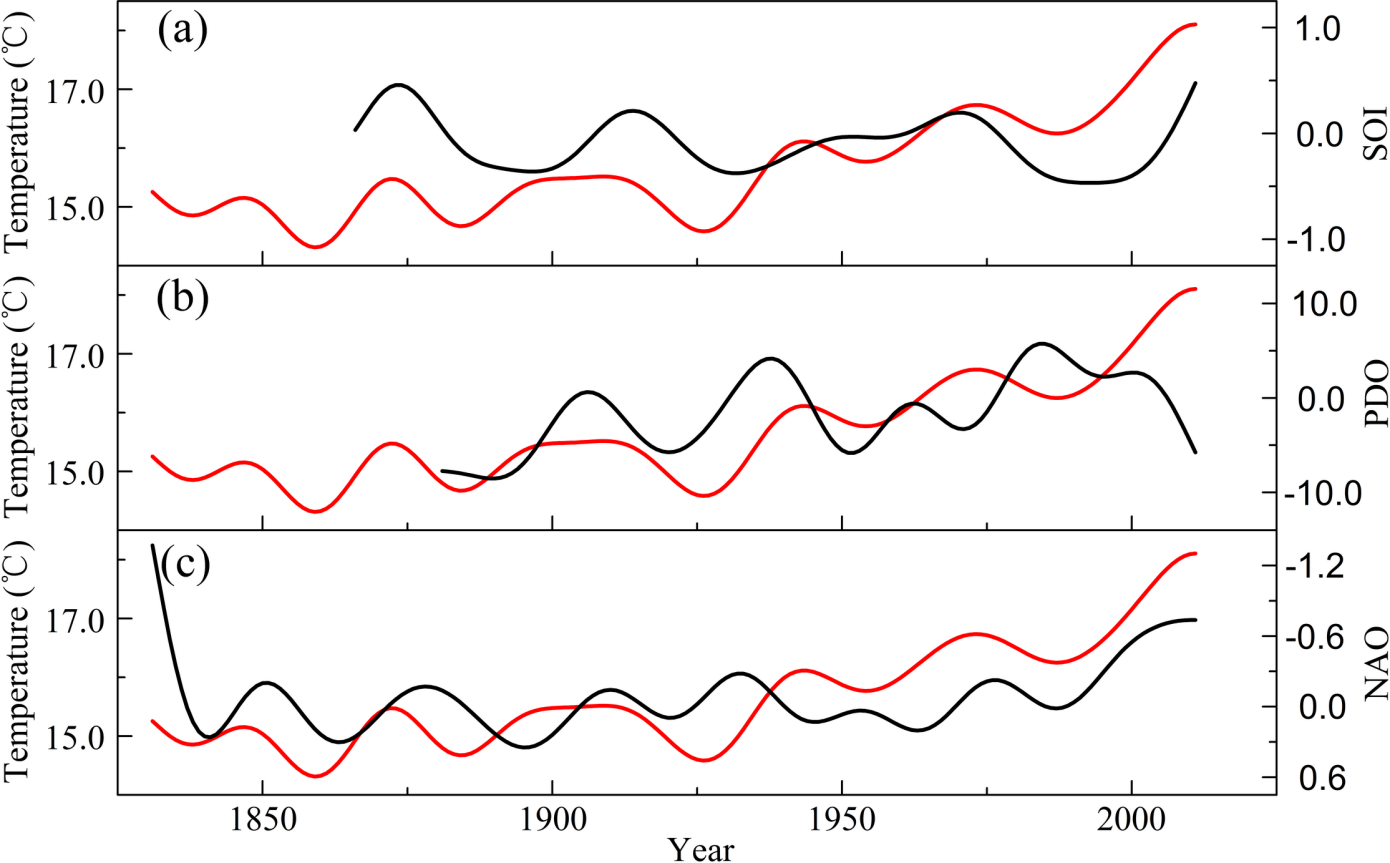
Comparison between our reconstructed temperature and the large-scale atmospheric-oceanic circulations (SO, PDO and NAO). These were smoothed using a 20-year low-pass filter.

It was reported that the SO had effects on the temperature variations in China [57]. By comparing the temperature history with August–September observations of the Southern Oscillation Index (SOI) series, we found that the two series had a significant relationship following the application of a 20-year low-pass filter (Fig 8). In addition to this, the 2.3–2.4-year, 2.8-year, 3.4–3.6-year and 5.0-year cycles of our temperature reconstruction also existed in the SOI [58]. These results might imply possible impacts of the SO on temperature in our study region, and the ring width of Qinghai spruce on Mt. Dongda may contain an SO signal. During a strong negative-phase of SO with El Niño developing, an anomalous descending motion appeared in the western Pacific [59], which could cause a large variability in the East Asian Summer Monsoon (EASM). The anomalous cyclonic circulation (with strong anomalous westerly winds) over the western Pacific reflected a weak western Pacific sub-tropical high in the El Niño developing summer. This resulted in the weakened EASM, which does not benefit the temperature in Northwest China [60].

The cycles at 9.9-year and 27.0-year may correspond to the PDO and NAO [61, 62], because there were also significant correlations between the temperature and the PDO and NAO. The correlation coefficient between the reconstructed temperature and the observed PDO index from the previous October to the present March at an interannual scale is 0.27 (p<0.005, 1881–2011). The correlation coefficient between the reconstructed temperature and the April–September NAO index is -0.20 (p<0.01, 1831–2011). In addition, after application of a 20-year low-pass filter, our temperature fluctuates synchronously with PDO and inversely with NAO at most time intervals. Moreover, these cycles appear widely in the tree-ring record of Northwest China [63, 64]. This may suggest that the temperature variability in Northwest China is partly influenced by large-scale circulation features, such as the NAO and PDO. The above analysis indicates that the effect of SO and NAO on the temperature of Northwest China is seasonally similar and the influence of PDO is seven months ahead. Previous work [59] showed that during a warm PDO phase in the winter, the Mongolian High strengthened and the Siberian High was weakened, which indicated an interdecadal seesaw-like oscillation between the two pressure systems. This pattern implied that the cold wind from Siberia tended to be weaker, and the air temperature was higher in Northwest China [65, 66]. A positive (negative) summer (July–September) NAO (SNAO) leads to a strong lower-level divergence (convergence) over the Asian jet entrance region, which in turn excites a strong upper-level convergence (divergence) driven by Ekman pumping [67, 68]. Such a convergence (divergence) then stimulates a zonally oriented quasi-stationary barotropic Rossby wave train along the Asian upper-level jet. Therefore, the SNAO signal is transported eastward to East Asia, resulting in an anomalous summer air temperature over Northwest China [69].

### Linkages with volcanic eruptions

Large volcanic eruptions inject sulfur gases into the stratosphere, which are converted to sulfate aerosols with an e-folding residence timescale of approximately one year. The climate response to large eruptions lasts for several years [70]. These phenomena can be recorded by reconstructed temperature series based on tree rings [71, 72]. We employed a historical/geological record based on the volcanic explosivity index (VEI) [73] that accurately records volcanic eruptions. The VEI value for each volcanic event was obtained from http://www.volcano.si.edu/search_eruption_results.cfm.

**Fig 9:**
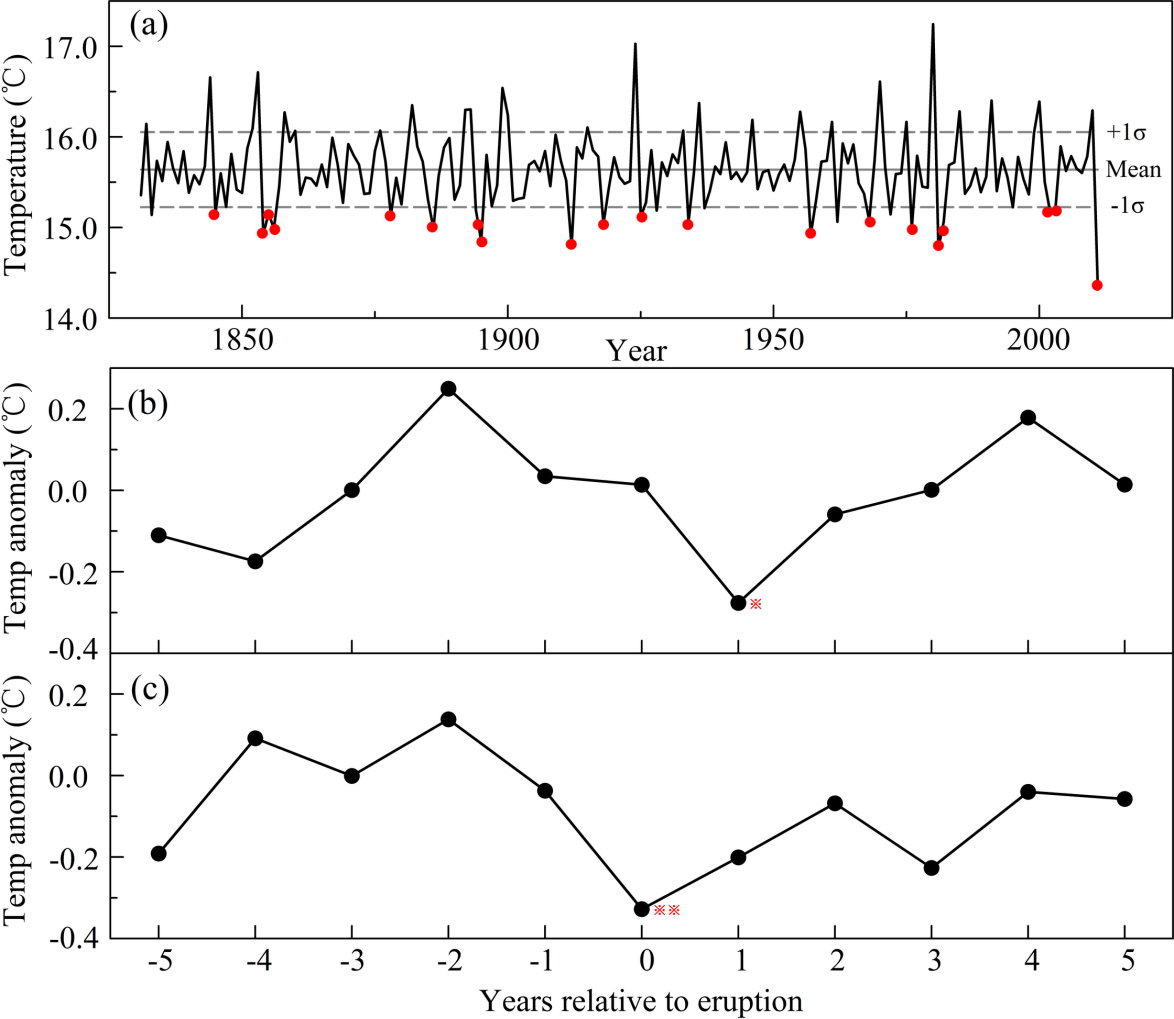
The connection between reconstructed temperature, major volcanic eruptions and the most negative ENSO years. (a) The corresponding relationship between the reconstructed temperature filtered using a 10-year high-pass filter and volcanic eruptions. The solid horizontal line represents the long-term mean of the filtered temperature for the period of 1831–2011; the dashed horizontal lines represent the mean value ± 1σ. The solid cycles indicate a cold year following a volcanic eruption event. (b) The filtered temperature superposed epoch analysis (SEA) [40] results for (b) the major volcanic eruptions (VEI >4) and (c) the most negative ENSO years [75] since 1831. One and two asterisks denoted anomalies significant at the 0.1 and 0.05 levels, respectively, based on the Monte Carlo test [41].

Before assessing the possible relationship, the reconstructed temperature was processed using a 10-year high-pass filter to highlight the interannual variability. The comparison of our reconstruction with large eruptions (VEI ≥4) revealed that the Mt. Dongda temperature decreased in association with volcanic eruptions. The 26 cold years, which were defined as temperature <mean−1σ, almost all occurred after volcanic eruptions (Fig 9A). To clarify this point further, we used the SEA method [40] to assess the relationship between the volcanic eruptions (VEI >4) and the reconstructed temperature for the past 181 years. Through simple compositing, the SEA method involves sorting data into categories dependent on a ‘key-date’ for synchronization and then comparing the means of those categories. Given sufficient data, a common underlying (causal) response to a forcing event should theoretically emerge in the average (composite) while other noise in the data should be cancelled [40]. The annual composites of the SEA results in the year +1 passed the 90% significant level test based on the Monte Carlo test [41]. The results indicated that anomalous cooling on Mt. Dongda occurred in one year after eruption (Fig 9B). The radiative effects of this aerosol cloud resulting from major eruptions can induce substantial global cooling, which may influence regional temperature change by atmospheric circulation [70]. Using observed data, it was previously found that large explosive volcanic eruptions could result in a decrease in surface temperature over almost all parts of China [74], which had a time lag of approximately one year. In general, major volcanic eruptions play an important role in high-frequency temperature change in Northwest China.

The effects of volcanic eruptions on climate change were possibly via large-scale atmospheric-oceanic variations [59]. It has been previously found that volcanic eruptions had directly impact on the El Niño/Southern Oscillation (ENSO) variation [75]. In order to clearly explain the relationships among volcanic eruptions, ENSO and temperature change in Northwest China, the SEA method was also used to analyze the relationships between temperature variation and the most negative ENSO years since 1831 [75], where the most negative ENSO years were those with values lower than the mean−1σ of the whole ENSO series. The results showed that anomalous cooling occurred in the year with more negative ENSO (Fig 9C). The reconstructed ENSO was the previous November–January Niño3.4 index and immediate cooling tended to occur in the Niño3.4 Pacific region in the year of eruption [75]. Combined with the SEA results (Fig 9B and 9C), we inferred that volcanic eruption influenced the Niño3.4 index from the November of the eruption year to the next January which affected the following May–October temperature of Northwest China; therefore, anomalous cooling occurred in one year after eruption.

## Conclusions

The May–October mean temperature history since 1831 was reconstructed based on tree-ring width chronology of Qinghai spruce from Mt. Dongda, which is located to the North of the Hexi Corridor in Northwest China. This reconstruction accounted for 46.6% of the variance of the observed temperature over the period of 1951–2011, and the transform function was verified as stable and reliable. During the reconstruction history from 1831 to 2011, there were 27 warm and 29 cold years. The entire reconstructed temperature series presented four warm periods (1890–1893, 1902–1907, 1935–1949 and 1953–2011) and four cold periods (1831–1889, 1894–1901, 1908–1934 and 1950–1952) at a low-frequency change. The longest warm and cold periods occurred during 1953–2011 and 1831–1889, respectively. Through comparison with the east-central Tibetan Plateau temperature series reconstructed using Wulan-Dulan tree rings, we found the two series presented similar warm/cold variations in some periods, indicating that the temperature variation in this study is a reliable proxy for Northwest China. Furthermore, our reconstruction also has good consistency with the NH temperature at a low-frequency change. Spectral analysis showed that the temperature variation in this region had significant periodicities at 2.3–2.4-year, 2.8-year, 3.4–3.6-year, 5.0-year, 9.9-year and 27.0-year, which are related to the SO, PDO or NAO. Combining the correlation and comparison analysis, it is suggested that the SO, PDO and NAO might partly influence the temperature variation in the Hexi Corridor of Northwest China, especially the low-frequency change. Almost all cold years in the high-pass-filtered temperature reconstruction coincided with major volcanic eruptions. These eruptions promoted anomalous cooling in the study region. The preliminary results presented in this paper provide new information on the long-term temperature changes in the Hexi Corridor of Northwest China and demonstrate the effects of large-scale atmospheric-oceanic circulation on climate change in Northwest China.

## Supporting Information

S1 Text Temperature reconstruction for the Mt. Dongda region, Northwest China. The data includes the reconstructed May–October mean temperature series.
(TXT)
